# Carbon Monoxide as a Molecular Modulator of Ischemia–Reperfusion Injury: New Insights for Translational Application in Organ Transplantation

**DOI:** 10.3390/ijms26167825

**Published:** 2025-08-13

**Authors:** Zhouyu Li, Kazuhiro Takeuchi, Yuichi Ariyoshi, Akira Kondo, Takehiro Iwanaga, Yurika Ichinari, Akiyuki Iwamoto, Kenya Shimizu, Kohei Miura, Shiori Miura, Lina Ma, Mitsuhiro Sekijima, Masayoshi Okumi, Hisashi Sahara

**Affiliations:** 1 Division of Experimental Large Animal Research, Life Science and Laboratory Animal Research Unit, Center for Advanced Science Research and Promotion, Kagoshima University, 8-35-1 Sakuragaoka, Kagoshima 890-8520, Japan; lizhouyu0519@gmail.com (Z.L.); takekazu04044@yahoo.co.jp (K.T.); tiwanaga181@gmail.com (T.I.);; 2 Transplantation Center, The Third Xiangya Hospital of Central South University, Changsha 410013, China; 3 Department of Nephrology, Kitasato University School of Medicine, Sagamihara 2520375, Japan; 4 Department of Urology, Kyoto Prefectural University of Medicine, Kyoto 6028566, Japan; okumi@koto.kpu-m.ac.jp; 5 Division of Digestive and General Surgery, Niigata University Graduate School of Medical and Dental Sciences, Niigata 9518520, Japan; 6 Department of Research Ethics and Biorisk Management, Institute for Research Administration, Niigata University, Niigata 9502181, Japan

**Keywords:** carbon monoxide, ischemia-reperfusion injury, gasotransmitter, anti-inflammatory signaling, cytoprotection, organ transplantation, carbon monoxide-releasing molecules, translational medicine, porcine

## Abstract

Carbon monoxide (CO) is generally recognized as a toxic gas; however, it has recently been identified as an endogenous gasotransmitter with significant cytoprotective properties. CO modulates key molecular pathways, including anti-inflammatory, anti-apoptotic, antioxidant, and vasodilatory signaling pathways, by targeting heme- and non-heme-containing proteins. These proteins include soluble guanylate cyclase, cytochrome P450 enzymes, MAPKs, and NLRP3. This review summarizes recent advances in understanding the molecular mechanisms associated with the protective effects of CO, particularly in the context of ischemia–reperfusion injury relevant to organ transplantation. We discuss preclinical data from rodent and large animal models, as well as therapeutic delivery strategies, such as inhalation, CO-releasing molecules, and gas-entrapping materials. We also reviewed early-phase clinical trials. The objective of this review is to provide a thorough exploration of CO as a potential therapeutic gas, with special emphasis on its application in transplantation.

## 1. Introduction

Organ transplantation is the definitive treatment for end-stage organ failure; however, the global shortage of donor organs poses a critical challenge. To address this issue, donation after circulatory death (DCD) has become an important strategy for expanding the donor pool for transplantation procedures. However, DCD organs are susceptible to prolonged warm ischemia and suboptimal procurement, resulting in inferior graft viability compared to organs from living or brain-dead donors [1,2]. As the clinical use of DCD grafts continues to increase, strategies to improve their quality are urgently needed. In this context, reducing ischemia–reperfusion injury (IRI) is essential for improving patient outcomes.

Carbon monoxide (CO), which has long been regarded as a toxic gas, has recently been recognized as a gaseous signaling molecule involved in essential cellular pathways [3]. Its anti-inflammatory, anti-apoptotic, and vasoregulatory properties suggest a potential role in mitigating IRI during transplantation. Our research focuses on the therapeutic use of CO as a medical gas. The growing interest in this field supports the promise of CO as a novel strategy for transplantation [4].

## 2. Toxicity of CO

CO is a colorless, odorless, tasteless, and flammable gas generated as a toxic byproduct of the incomplete combustion or oxidation of organic materials. Owing to its amphiphilic nature, CO readily diffuses across lipid bilayers without specific transporters or receptors [5]. It binds to hemoglobin with 200–250 times the affinity of oxygen, forming carboxyhemoglobin (COHb), which reduces oxygen delivery and causes hypoxia [6,7]. Clinical symptoms such as dizziness, dyspnea, and headache may occur when the COHb level exceeds 20%. Higher levels (50–80%) can lead to neurotoxicity, cognitive dysfunction, and death [6,7].

In addition to its effects on hemoglobin, CO directly interacts with intracellular heme-containing proteins in the mitochondrial electron transport chain, including cytochrome c and cytochrome c oxidase (CytOx). This interaction inhibits adenosine triphosphate (ATP) production and increases the generation of reactive oxygen species (ROS), contributing to mitochondrial dysfunction, a key mechanism underlying acute CO poisoning [8,9]. Furthermore, CO binding to non-mitochondrial heme proteins, such as myoglobin, leads to cardiac and skeletal muscle injury. CO toxicity is mediated through multiple pathways beyond heme–protein interactions [10,11].

## 3. Endogenous CO Production Mechanism

**Figure 1:**
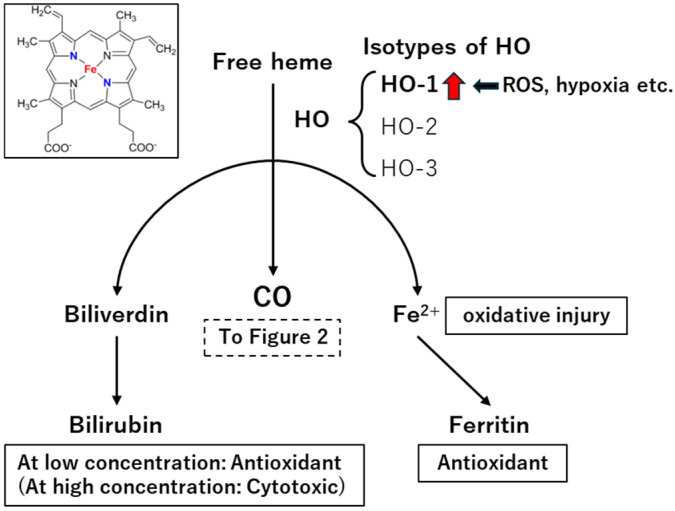
Endogenous production of carbon monoxide (CO) by heme oxygenase (HO). HO-1 catalyzes the degradation of heme into biliverdin, Fe^2+^, and CO. This enzymatic reaction is a major endogenous source of CO in mammalian cells. HO-1 expression is often upregulated (red arrow) under conditions such as oxidative stress, hypoxia, and other cellular stressors. ROS: reactive oxygen species.

CO is endogenously generated through the enzymatic degradation of heme by heme oxygenase (HO), which produces biliverdin, ferrous iron (Fe^2+^), and CO as byproducts [4,12]. Heme, a key component of hemoproteins such as hemoglobin, myoglobin, and cytochromes, is released during oxidative stress induced by ischemia, neutrophil activation, and other pathological stimuli. Free heme, being lipid-soluble, readily integrates into cell membranes and promotes cytotoxicity via enhanced ROS production (Figure 1).

HO enzymes counteract this toxicity by degrading free heme. There are three isoforms of HO: HO-1, HO-2, and HO-3. HO-1 is inducible and strongly upregulated in response to various stressors, including heavy metals, endotoxins, ultraviolet radiation, ROS, hypoxia, and heat shock [13]. In contrast, HO-2 is constitutively expressed in most tissues, particularly in the brain and testis [14]. HO-3 is considered a pseudogene derived from the HO-2 transcript, with no clearly defined enzymatic activity [15].

In addition to CO generation, HO enzymes regulate multiple physiological processes, including respiration, blood pressure, signal transduction, neuroprotection, and apoptotic pathways [14]. In addition, other heme degradation products modulate oxidative stress. For example, biliverdin and its reduced form, bilirubin, exhibit antioxidant effects at low concentrations but can become cytotoxic at higher concentrations [16]. Similarly, excess Fe^2+^ promotes oxidative injury, which is mitigated by ferritin-mediated iron sequestration [17].

## 4. Target Proteins Mediating the Cytoprotective Effects of CO

**Figure 2:**
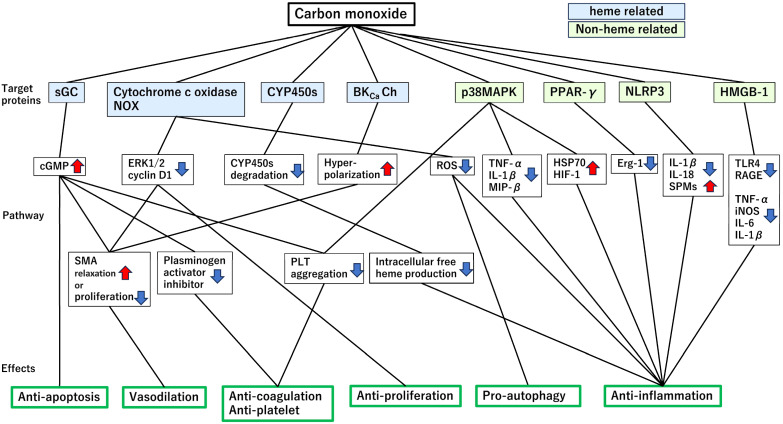
Carbon monoxide (CO) target proteins and pathways exert protective effects. Mechanisms of CO-mediated anti-apoptosis, vasodilation, anticoagulation, and pro-autophagy effects through heme-related and non-heme-related target proteins. The diagram illustrates how CO exerts multiple biological effects by interacting with both heme-related targets (e.g., sGC and cytochrome c oxidase NOX) and non-heme-related proteins (e.g., p38 MAPK and PPAR-γ). The elements are divided into three sections according to their primary binding proteins, pathways, and effects. Heme-related proteins are highlighted with a blue background, whereas non-heme-related proteins are marked with a green one. Within the pathways, upregulation is indicated by upward red arrows and downregulation by downward blue arrows. The effects are depicted by directly connecting pathway lines to boxes representing the respective biological outcomes. BKcaCh: large-conductance Ca^2+^-activated potassium; cGMP: cyclic guanosine monophosphate; CYP450s: cyto-chrome P450 enzymes; Erg-1: early growth response 1; ERK1/2: extracellular signal-regulated kinase 1/2; HIF-1: hypoxia-inducible factor 1; HSP70: heat shock protein 70; HMGB1: high-mobility group box 1; iNOS: FNOX: NAD(P)H oxidase; NLRP3: NLR family pyrin domain containing 3; p38MAPK: p38 mitogen-activated protein kinase; PPAR-γ: peroxisome proliferator-activated receptor-γ; PLT: platelet; RAGE: receptor for advanced glycation end-products; ROS: reactive oxygen species; SMA: smooth muscle actin; sGC: soluble guanylate cyclase.

The cytoprotective effects of CO are largely mediated through interactions with heme-containing proteins, over 25 of which have been identified as molecular targets [7,18]. These proteins play diverse roles in anti-inflammatory, anti-apoptotic, anti-proliferative, anticoagulant, pro-autophagic, and vasoregulatory pathways. The key representatives are described below and illustrated in Figure 2.

### 4.1. Heme-Containing Proteins

#### 4.1.1. Soluble Guanylate Cyclase (sGC)

sGC is a cytosolic heme-containing enzyme that catalyzes the conversion of guanosine triphosphate (GTP) to cyclic guanosine monophosphate (cGMP), a second messenger involved in vasodilation, inhibition of platelet aggregation, fibrinolysis, and suppression of smooth muscle proliferation [19,20,21,22,23,24]. CO activates sGC via direct heme binding, leading to increased intracellular cGMP levels and cytoprotective downstream effects. However, the dissociation constant (Kd) of CO for sGC (~240 μM) is significantly weaker than that for hemoglobin (0.7 nM–4.5 μM) or myoglobin (29 nM), raising questions regarding the physiological relevance of sGC activation by CO in vivo [25]. Further studies are needed to clarify whether CO is transferred from high-affinity carriers, such as hemoglobin, to low-affinity targets, such as sGC under biologically relevant conditions.

#### 4.1.2. Cytochrome c Oxidase (CytOx) and Nicotinamide Adenine Dinucleotide Phosphate (NADPH) Reduced Form Oxidase

CytOx, the terminal enzyme of the mitochondrial respiratory chain, is inhibited by CO in a concentration-dependent manner, resulting in reduced ATP production, hyperpolarization of the mitochondrial membrane potential, and increased ROS generation [11,26]. These effects are particularly pronounced in hypoxia or inflammation. CO also inhibits NADPH oxidase (NOX) activity, thereby suppressing ERK1/2 phosphorylation and cyclin D1 expression, and ultimately reducing vascular smooth muscle cell proliferation [27]. In immune cells, such as mouse T cells, CO attenuates ROS production by inhibiting both NOX and the mitochondrial electron transport chain complexes I–IV [28]. Interestingly, low-dose CO (250 ppm) has been shown to induce autophagy in alveolar and bronchial epithelial cells by increasing mitochondrial ROS and upregulating the expression of autophagic protein microtubule-associated protein-1 light chain-3B (LC3B), contributing to cellular resilience against hyperoxia-induced injury [29].

#### 4.1.3. Cytochrome P450 Enzymes (CYP450s)

CYP450s are membrane-bound monooxygenases involved in xenobiotic metabolism, lipid processing, and endogenous signaling [30]. Under oxidative stress, these enzymes degrade and release free heme and iron, promoting ROS generation and causing tissue injury. CO binds to CYP450s, stabilizing their structure, preventing their degradation, and limiting heme release. In a kidney cold ischemia model, CO dissolved in UW solution preserved CYP450 integrity, reduced inflammation, and protected against IRI [31]. In malignant tissues, CYP3A4 and CYP2C8 are overexpressed and can inactivate chemotherapeutic agents such as paclitaxel. CO-mediated inhibition of these isoforms enhances drug efficacy and demonstrates a potential indirect anticancer effect [32].

#### 4.1.4. Large-Conductance Ca^2+^-Activated Potassium (BKCa) Channels

BKCa channels regulate membrane potential and vascular tone. They are inhibited by heme binding to their α-subunit; however, CO can reverse this inhibition by selectively interacting with reduced heme (Fe^2+^) and facilitating channel opening [33]. CO promotes BKCa activation through multiple mechanisms. In cerebral arterioles, it enhances Ca^2+^ spark-coupled activation, whereas in mesenteric arteries, it activates BKCa independently of Ca^2+^ or cGMP [34,35]. In human cardiac fibroblasts, CO increases BKCa current amplitude via pathways involving nitric oxide synthase (NOS), protein kinase G (PKG), protein kinase A (PKA), and S-nitrosylation [36]. These effects contribute to vasodilation and cardiovascular protection.

### 4.2. Non-Heme-Containing Proteins

#### 4.2.1. Mitogen-Activated Protein Kinases (MAPKs)

MAPKs are a family of serine/threonine kinases that transduce extracellular stress signals into cellular responses, particularly those related to inflammation and apoptosis. CO modulates the p38 MAPK pathway and suppresses the production of cytokines, including TNF-α, IL-1β, and macrophage inflammatory protein-1β (MIP-1β) [37]. CO also inhibits the coagulation cascade and platelet activation while promoting the expression of protective proteins, such as heat shock protein 70 (Hsp70) and hypoxia-inducible factor-1 (HIF-1), thereby enhancing cellular defense mechanisms against endotoxin-induced injury [38].

#### 4.2.2. Peroxisome Proliferator-Activated Receptor γ (PPARγ)

CO induces increased expression of the transcription factor PPARγ, resulting in the inhibition of the upregulation of early growth response-1 (Egr-1) expression caused by stress from the administration of mechanical ventilation. The application of CO prevented lung injury during ventilation, reduced the upregulation of stress-related genes, and decreased neutrophil infiltration in the lungs [39].

#### 4.2.3. Nucleotide-Binding Domain, Leucine-Rich Repeat-Containing Family, Pyrin Domain-Containing 3 (NLRP3) Inflammasome

The NLRP3 inflammasome is a key regulator of innate immunity that mediates the maturation and secretion of inflammatory cytokines such as IL-1β and IL-18. CO has been shown to directly and indirectly suppresses NLRP3 inflammasome activation. One mechanism involves the promotion of specialized pro-resolving mediators that help to resolve inflammation and may counteract the pro-inflammatory activity of NLRP3 [40]. Additionally, CO preserves mitochondrial integrity, thereby preventing the main triggers of inflammasome activation from occurring. In bone-marrow-derived macrophages stimulated with lipopolysaccharide (LPS) and ATP, CO reduced caspase-1 activation and the subsequent release of cytokines. It also inhibits mitochondrial ROS production, preserves mitochondrial membrane potential, and blocks the release of mitochondrial DNA into the cytosol, which is a known signal for NLRP3 activation [41].

#### 4.2.4. High-Mobility Group Box 1 (HMGB1)

HMGB1 is a nuclear DNA-binding protein that acts as a potent damage-associated molecular pattern (DAMP) when released extracellularly. CO inhibits the acetylation and translocation of HMGB1 by suppressing the activity of histone acetyltransferases. CO also activates sirtuin 1 (SIRT1), which deacetylates HMGB1, thereby limiting its release and reducing downstream inflammation. CO-releasing molecules (e.g., CORM-2) have been shown to reduce the expression of Toll-like receptor 4 (TLR4), receptor for advanced glycation end products (RAGE), and associated inflammatory mediators in hepatic and renal IRI models [42,43].

#### 4.2.5. Glycogen Synthase Kinase-3β (GSK3β)

GSK3β is a multifunctional serine/threonine kinase involved in cellular apoptosis and metabolism. In hepatic ischemia–reperfusion models, CO preserves the phosphorylated (inactive) form of GSK3β via activation of the phosphoinositide 3-kinase (PI3K)/Akt pathway. This protective effect is abrogated by PI3K inhibition, suggesting that this signaling axis plays a central role in CO-mediated cytoprotection [44].

## 5. Delivery Methods for Therapeutic CO

Following the discussion of molecular targets, the development of safe and effective delivery strategies is critical for translating CO-based therapies into the clinical setting. This section reviews the current and emerging delivery modalities, emphasizing the optimization of therapeutic efficacy and minimization of toxicity.

### 5.1. Inhalation of Gaseous CO

The most straightforward and reproducible method is the inhalation of gaseous CO. Inhaled concentrations can be precisely adjusted by the real-time monitoring of COHb levels [7]. Owing to CO’s inherent toxicity, strict safety protocols must be followed, including administration via tracheal intubation to ensure controlled delivery and reduce systemic adverse effects [7].

### 5.2. CO-Enriched Organ Preservation Solutions

Although CO is poorly soluble in water, it can be dissolved in organ preservation solutions, which is particularly relevant for transplantation. Immersion of grafts in CO-saturated fluid allows direct tissue exposure during cold storage and has been shown to reduce IRI [45]. However, further investigation is required to determine the optimal CO content and ensure consistent efficacy and safety in clinical settings [46].

### 5.3. CO-Releasing Molecules (CORMs)

CORMs are synthetic compounds engineered to release CO in vivo without significantly elevating systemic COHb concentrations [47,48]. These molecules are typically classified based on their metal cores: iron-based (e.g., CORM-307 and -308), manganese-based (e.g., CORM-1 and -401), and ruthenium-based (e.g., CORM-2 and -3). Non-metallic options, such as boron-based CORM-A1, offer slower CO release and improved biocompatibility than their metallic counterparts. Organic solvents such as methylene chloride have also been investigated as CO carriers [49]. Despite these advances, challenges remain regarding in vivo stability, release kinetics, and potential metal-associated toxicity [50]. Moreover, recent findings have highlighted the CO-independent activities of certain CORMs, such as catalase-like activity, thiol reactivity, and NAD(P)+ reduction, which may complicate the interpretation of their biological effects [51].

### 5.4. Gas-Entrapping Materials (GEMs)

GEMs are a novel class of delivery systems designed to physically encapsulate CO within Generally Recognized As Safe (GRAS) materials, such as foams, hydrogels, and solid matrices. These are administered via the gastrointestinal tract and allow non-inhalational CO delivery with precise dosing and minimal systemic toxicity [52]. This approach expands the potential routes for CO administration and offers improved patient safety and compliance.

## 6. Effectiveness of CO as a Therapeutic Agent for Transplant-Related and IRI, Organized by Organs

In this section, we performed a thorough search in PubMed using key terms related to CO, transplantation, and IRI, along with specific organs and animal models. For rodent models, we specifically focused on studies published before 2012, referencing the comprehensive review by Ozaki et al. [53] as a basis for selecting earlier data.

### 6.1. CO Application in Transplantation: Findings from Rodent Models

Rodent studies have provided critical insights into the mechanisms and therapeutic potential of CO in transplantation. A 2012 review summarized preclinical research on CO’s cytoprotective effects of CO in IRI [53]. Since then, significant advancements have been made, particularly in the development of organ-specific transplantation models. Here, we focus on post-2012 findings organized by organ type. In vitro and small-scale studies were excluded because of their limited clinical relevance to this review. The experimental conditions and major outcomes are summarized in Table 1 and Table 2.

#### 6.1.1. Heart

Rodent models have consistently demonstrated the cardioprotective effects of CO against IRI and experimental heart transplantation. Preconditioning with CO activates insulin signaling pathways and attenuates mitochondrial perturbations and oxidative stress [54,55]. These effects are thought to arise not only from the modulation of hypoxia-sensitive signaling pathways but also from CO’s intrinsic ability to inhibit cellular respiration, to which the heart is particularly sensitive. Together, these findings support the potent anti-apoptotic and mitochondrial protective actions of CO in cardiac IRI.

#### 6.1.2. Lung/Trachea

In rodent lung transplantation models, CO administration has been shown to reduce epithelial and subepithelial thickening, luminal obliteration, alveolar hemorrhage, immune cell infiltration, and fibrosis [61,62,63,64]. The inherent capacity of the lungs to hold gas enables high local CO concentrations and minimizes systemic toxicity. Most studies have applied CO as donor lung pretreatment, which resulted in both short-term benefits (e.g., reduced alveolar hemorrhage) and long-term improvements in airway remodeling and graft compliance [61,63]. These protective effects are mediated by CO’s anti-inflammatory, anti-apoptotic, and antioxidant properties of CO. Additionally, CO’s anti-lipid peroxidation activity contributes to the preservation of lung architecture and function post-transplantation.

#### 6.1.3. Kidney

CO exerts renoprotective effects via multiple mechanisms. It inhibits HMGB1 translocation by suppressing nuclear histone acetyltransferase activity, thereby attenuating inflammation [42]. Furthermore, CO modulates purinergic and circadian signaling, as evidenced by increased CD39 expression, decreased adenosine A1 receptor (Adora1) expression, upregulation of A2A/A2B receptors and the clock protein Per2, and increased erythropoietin levels [56]. Combination therapy with hydrogen and CO has shown synergistic effects, lowering blood urea nitrogen and inflammatory cytokine levels while improving oxidative stress responses [57,67]. Post-reperfusion CO treatment reduced serum creatinine, kidney injury molecule-1 (KIM-1), and tubular apoptosis, with transcriptomic alterations involving PPAR signaling [58]. In addition, CO suppresses renal fibrosis by inhibiting epithelial–mesenchymal transition and TGF-β1 signaling [59]. Notably, pretransplant exposure to high-pressure CO (2000 hPa) during cold storage attenuated early inflammation and apoptosis and significantly reduced interstitial fibrosis 100 d after transplantation [66]. These data highlight the potential of CO in protecting the kidneys from IRI and preventing chronic graft injury.

#### 6.1.4. Liver

In hepatic IRI models, CO preserved liver function by maintaining the phosphorylation of GSK3β, with evidence suggesting regulation via the PI3K/Akt pathway. Inhibition of PI3K abolished this protective effect, indicating its critical involvement [44]. CO also upregulates SIRT1 expression by suppressing miR-34a, promoting the deacetylation of p65 and p53, and conferring anti-inflammatory and anti-apoptotic effects [60]. CORM-2 pretreatment further enhances SIRT1-mediated deacetylation of HMGB1, preventing its nuclear export and release, thereby mitigating hepatic damage [43]. These studies collectively demonstrate that CO protects the liver from IRI through multiple signaling pathways converging on mitochondrial preservation and regulation of inflammation-related transcription factors.

### 6.2. Applications of CO in Experimental Evaluations Based on Non-Transplant Porcine Models

Preclinical studies using non-transplant pig models have demonstrated the protective effects of CO in multiple organ systems. These models provide mechanistic insights into both the systemic and localized actions of CO, particularly its ability to modulate inflammation, preserve energy metabolism, and promote recovery after ischemic injury. Collectively, these findings highlight the therapeutic potential of CO, beyond transplantation.

#### 6.2.1. Heart

In a porcine model of hemorrhagic shock, inhalation of low-dose CO (250 ppm) preserved mitochondrial respiratory function in the intestinal tissue, as evidenced by a maintained respiratory control ratio following resuscitation. These findings suggest that CO limits oxidative stress by preserving mitochondrial bioenergetics during systemic recovery from severe hypoperfusion [68].

#### 6.2.2. Lung

In a swine sepsis model induced by LPS, CO inhalation (250 ppm, 1 h) improved pulmonary gas exchange, suppressed systemic inflammation by decreasing IL-1β levels, and elevated anti-inflammatory IL-10 levels [69]. In another study using a CPB-induced lung injury model, CO preconditioning (250 ppm, 1 h) significantly downregulated pro-inflammatory cytokines (TNF-α, IL-1β) and upregulated IL-10 in lung tissue [70]. Notably, this anti-inflammatory effect was abolished by quercetin, a nonspecific inhibitor of heat shock proteins (HSPs), suggesting that HSPs mediate CO’s protective role of CO [71].

#### 6.2.3. Intestine

In a model of abdominal surgery, preoperative inhalation of CO (250 ppm for 3 h) significantly attenuated postoperative ileus in pigs. CO-treated animals exhibited increased contractility of the intestinal circular muscle in vitro and improved gastrointestinal transit in vivo. These effects were achieved without compromising animal safety, as the vital signs remained stable and COHb levels were within the acceptable limits [72].

### 6.3. Application of CO in the Transplantation Field Based on Porcine Model

Organ transplantation inevitably involves ischemia and hypoxia during the interval between graft procurement and reperfusion, resulting in IRI [73,74]. IRI is characterized by ATP depletion, hypoxanthine accumulation, ROS production, and the release of pro-inflammatory cytokines and DAMPs, ultimately leading to inflammatory cascades, cellular apoptosis, and necrosis [75,76,77].

In large animal transplantation models [4,49,53,78,79], including lung [80], kidney [81,82,83], and heart [84] transplantation, CO has demonstrated cytoprotective effects mediated by anti-inflammatory, anti-apoptotic, vasodilatory, and antioxidant mechanisms. Given their close physiological resemblance to humans, pig models are especially valuable for evaluating the translational potential of CO therapy in humans. Table 3 and Table 4 summarize the representative studies.

#### 6.3.1. Heart

In a porcine cardiopulmonary bypass (CPB) model, CO preconditioning (250 ppm for 2 h) enhanced myocardial energy stores (ATP and phosphocreatine), reduced interstitial edema and cardiomyocyte apoptosis, and facilitated hemodynamic recovery with fewer defibrillations required after reperfusion [85]. In contrast, studies using inhaled CO in myocardial IRI models have shown inconsistent protective effects, likely due to subtherapeutic CO concentrations and insufficient observation periods [86,87]. Notably, intravenous administration of CORM-A1 (4.27 mM) resulted in significant reductions in infarct size and myocardial injury markers and improved left ventricular function, suggesting the anti-inflammatory and anti-proliferative properties of CO [88].

#### 6.3.2. Lung

In a porcine pulmonary IRI model involving 90 min vascular and bronchial occlusion, CO inhalation (250 ppm for 360 min) significantly improved arterial oxygenation and suppressed histopathological injury, including alveolar edema, hemorrhage, neutrophil infiltration, and endothelial damage. Serum IL-1β and IL-6 levels were also reduced [89].

In fully MHC-mismatched lung transplantation using miniature swine, perioperative CO inhalation (200–250 ppm to both donor and recipient) preserved graft function in four of five recipients and suppressed anti-donor IgG production despite tacrolimus monotherapy [80].

In a pig-to-cynomolgus monkey xenogeneic lung transplant model, CO reduced inflammatory cell infiltration, thrombosis, and inflammatory cytokine expression while preserving platelet counts and increasing HO-1-positive cell infiltration, although overall graft survival was not prolonged [95].

Additionally, in a CPB-induced lung IRI model, CO inhalation (250 ppm for 60 min) downregulated TNF-α and IL-6, upregulated HSP70 and IL-10, suppressed caspase-3 activity, and attenuated alveolar damage and leukocyte infiltration [90].

#### 6.3.3. Kidney

In a DCD model, low-dose CORM-3 (50–100 μM) significantly improved renal blood flow, creatinine clearance, and urine output [91]. Pretreatment with 50 μM CORM-3 also stabilized renal function and suppressed serum creatinine elevation [92].

In an ex vivo perfusion model, CORM-401 (200 μM) administered after 4 h of cold storage reduced vascular resistance, apoptosis, and necrosis, which was associated with the downregulation of TLR2/4/6 [93].

In an autologous renal Tx model, grafts preserved for 48 h in CO-saturated UW solution exhibited reduced histologic damage and lower expression of IL-1β, IL-6, IL-18, TGF-β, and phosphorylated Smad3 at 3 h and 14 d post-reperfusion [97].

In a delayed graft function model, 60 min CO inhalation improved renal function recovery within 7 days, reduced tubular necrosis and apoptosis, downregulated tissue factor, P-selectin, MCP-1, and HSPs, and promoted tubular regeneration [96].

#### 6.3.4. Liver

In a hepatic IRI model with 45 min occlusion of portal vein and native hepatic artery occlusion, CO inhalation at 250 ppm for 345 min significantly reduced serum liver enzyme elevation and histological damage, including congestion, degeneration, necrosis, and neutrophil infiltration, while inflammatory cytokines (TNF-α, HMGB1, IL-6) were markedly suppressed. These injuries were fully resolved by day 4 after reperfusion [94].

Notable, based on the findings of long-term studies conducted in large animal models over periods of 30 days or more, no significant side effects of CO administration were observed [80,89,94]. This supports the safety of CO as a therapeutic agent in transplantation models, providing further assurance for its potential clinical application.

## 7. Application of CO in Clinical Research

Clinical trials have primarily focused on evaluating the safety, feasibility, and preliminary efficacy of CO inhalation in patients with pulmonary diseases. In a Phase I trial involving patients with acute respiratory distress syndrome (ARDS) secondary to sepsis, low-dose CO (100 or 200 ppm for 1 h daily over 5 days) was well tolerated, with COHb levels maintained below 10% and no major adverse events observed. Among the inflammatory markers, circulating mitochondrial DNA was significantly reduced, although the levels of IL-18 and RIPK3 remained unchanged [98]. A subsequent Phase IIa study examined the effects of CO inhalation (100–200 ppm, twice daily for 12 weeks) in patients with idiopathic progressive fibrosing interstitial lung disease. While no significant improvements were found in serum matrix metalloproteinase-7 (MMP-7), pulmonary function, or disease severity, CO treatment was well tolerated without any adverse events [99]. These findings confirm the clinical feasibility and safety of low-dose CO inhalation, even in patients with acute or chronic pulmonary pathologies.

In the field of transplantation, the clinical application of CO remains limited but promising. A pilot study of human islet transplantation evaluated the ex vivo exposure of donor islets to 1% CO gas bubbled into the culture medium for 3–4 h during the isolation process. CO-treated islets demonstrated increased viability, reduced β-cell death, suppressed CCL23, and enhanced CXCL12 expression on days 1 and 3 after transplantation. No adverse effects were observed during the six-month follow-up period, underscoring the safety of CO preconditioning in cellular transplantation [100]. Despite these encouraging outcomes, critical challenges, such as defining optimal dosing, delivery methods, and long-term risks, must be addressed before broader clinical applications. Nonetheless, the convergence of data from rodent and swine models, together with early-phase human studies, supports the translational potential of CO therapy in both transplantation and critical care.

## 8. Comparison Between CO and Other Gaseous Signaling Molecules

In addition to carbon monoxide (CO), other endogenously produced gaseous signaling molecules, such as nitric oxide (NO) and hydrogen sulfide (H_2_S), have attracted attention for their therapeutic potentials. These gases share pleiotropic effects, including anti-inflammatory, anti-apoptotic, antioxidant, and cytoprotective properties, but differ markedly in their biochemical properties, synthesis pathways, and clinical applications [101]. Table 5 summarizes the key features of the gases.

NO is synthesized from L-arginine via NOS isoforms, such as endothelial NOS (eNOS) and neuronal NOS (nNOS), producing NO through a five-electron oxidation reaction in the presence of oxygen [102]. NO is clinically approved for inhalation therapy, particularly for pulmonary arterial hypertension and ARDS [103], owing to its vasodilatory and anti-inflammatory effects. NO can be delivered via direct inhalation or the administration of NO-donating compounds, such as organic nitrates, metal complexes, and diazeniumdiolates [104]. Because the direct measurement of NO is difficult, surrogate markers such as nitrite and nitrate (NOx) are used, which are typically measured via chemiluminescence- or fluorescence-based assays [105]. In transplantation, reduced NO levels in renal allograft recipients have been linked to worse outcomes, supporting the potential role of NO in modulating graft health [106].

H_2_S is enzymatically produced from L-cysteine by cystathionine β-synthase (CBS) and cystathionine γ-lyase (CSE) [107]. Although toxic at high concentrations, H_2_S exhibits dose-dependent cytoprotective effects. Its therapeutic potential has been explored in multiple systems, including the kidneys (attenuation of fibrosis) [108], nervous system (neuroprotection and cognitive enhancement) [109], and cardiovascular system (protection against ischemic injury and heart failure) [110]. Due to safety concerns regarding inhalation, H_2_S is typically delivered using donor compounds that are administered intravenously [111]. The measurement of H_2_S concentration is commonly performed using spectrophotometric and ion-selective electrode techniques.

Although CO, NO, and H_2_S have similar biological functions, differences in pharmacokinetics, toxicity thresholds, and delivery systems must be considered when evaluating their clinical applicability. Furthermore, the synergistic effects of H_2_S and CO interfere with the NO production pathway by inhibiting iNOS and play a crucial role in reducing IRI [79]. Their combined application may further enhance these protective effects. Further studies are needed to delineate the optimal administration strategies for each gas and define organ- or disease-specific indications.

## 9. Conclusions

CO, once considered solely toxic, is now recognized as a biological signaling molecule with therapeutic potential. Preclinical studies in ischemia–reperfusion injury and transplantation models have demonstrated its anti-inflammatory, anti-apoptotic, and cytoprotective effects, as well as the benefits of various delivery strategies. Large animal experiments have shown that low-dose CO improves graft function, preserves tissue architecture, and enhances survival in various organs. Emerging strategies for clinical translation include localized CO delivery to donor organs during cold storage or normothermic machine perfusion, which may enhance graft protection while minimizing systemic CO exposure. Despite these encouraging results, challenges remain, including the molecular basis by which transient CO exposure leads to durable immune modulation and graft protection, which requires further investigation. Supported by preclinical and early clinical evidence, CO-based therapy represents a promising adjunct to transplantation.

## Figures and Tables

**Table 1:** Application of CO in the transplantation field based on rodent IRI model.

Author (Year)	Target Organ	Animal: Model	Observation Period	Delivery Method	Administration Timing	Main Effects	Ref.
Zhang (2021)	Heart	Rat: 30 min ischemia	120 min	CO-PolyPHb 0.5 g Hb/kg/d IV	From 3 d before ischemia	Cardioprotection via improved mitochondrial function and activation of the insulin signaling pathway	[54]
Kumar (2021)	Heart	Rat: 30 min ischemia	90 min	CORM-2 20 μmol/L Perfusion	For 10 min before ischemia	Cardioprotection via improved mitochondrial function and reduced oxidative stress	[55]
Ruan (2014)	Kidney	Mice: 50 min ischemia	14 d	CORM-2 20 mg/kg IV	At 1 h before ischemia	Renoprotection and prolonged survival via inhibition of ischemia-induced HMGB1 expression and suppression of inflammatory cytokine	[42]
Correa-Costa (2018)	Kidney	Mice: 45 min ischemia	24 h	CO gas 250 ppm Inhalation	For 1 h before ischemia	Renoprotection via upregulation of anti-inflammatory CD39 and Adora2a/2b	[56]
Nishida (2018)	Kidney	Rat: 45 min ischemia	24 h	CO + H_2_ gas CO 250 ppm Inhalation	For 24 h from 15 min before reperfusion	Renoprotection via enhanced superoxide radical scavenging activity and inhibition of inflammatory cytokine upregulation	[57]
Kim (2020)	Kidney	Rat: 75 min ischemia	24 h	CORM-3 10 mg/kg IV	At 1 h before ischemia	Renoprotection via reduction in apoptotic renal tubular cells and prevention of downregulation of PPAR signaling-related gene	[58]
Nagasaki (2022)	Kidney	Mice: 35 min ischemia	14 d	CO enrich-RBC 700 mgHb/kg IV	At 1, 3, and 5 d after ischemia	Less renal fibrosis via the suppression of epithelial–mesenchymal transition and transforming growth factor-β1 secretion	[59]
Kim (2013)	Liver	Mice: 90 min ischemia	6 h	CO gas 250 ppm Inhalation	For 12 h before ischemia	Hepatoprotection via maintenance of GSK3β phosphorylation	[44]
Kim (2015)	Liver	Mice: 60 min ischemia	6 h	CO gas 250 ppm Inhalation	For 12 h before ischemia	Hepatoprotection via inhibition of miR-34a/SIRT1 pathway.	[60]

Abbreviations: Adora: adenosine receptor A; CD: cluster of differentiation; CORM: carbon monoxide-releasing molecules; CO: carbon monoxide; d: day/days; GSK3β: glycogen synthase kinase 3β; h: hour/hours; Hb: hemoglobin; H_2_: hydrogen; HIF-1α: hypoxia-inducible factor 1-alpha; HMGB1: high-mobility group box 1; IRI: ischemia–reperfusion injury; IV: intravenous; miR-34a: microRNA-34a; Nrf2: Nuclear factor erythroid 2-related factor 2; PPAR: peroxisome proliferator-activated receptor; PolyPHb: polymerized human placenta hemoglobin; RBC: red blood cell; SIRT1: sirtuin 1; min: minutes.

**Table 2:** Application of CO in the transplantation field based on rodent transplant model.

Author (Year)	Target Organ	Animal: Model	Observation Period	Delivery Method	Donor CO	Recipient CO	Main Effects	Ref.
Ohtsuka (2014)	Trachea	Mice: Ortho and Hetero	Ortho: 7 d Hetero: 21 d	CORM-2 10 mg/kg IP	No	At 1 h before Tx, then every 3 d	Less thickening in epithelial and subepithelial airway layers and obliteration with less inflammatory cell infiltration and lower inflammatory cytokines	[61]
Meng (2016)	Lung	Rat: Ortho	3 h	Perfusion 500 ppm	3 h after procurement	No	Less graft injury via anti-inflammatory, antioxidant, and anti-apoptosis effects	[62]
Fujiwara (2019)	Lung	Rat: Ortho	90 min	High-pressure chamber 1.5 atm	24 h after procurement	No	Less graft injury with lower inflammatory mediator and lactic acid levels	[63]
Aoki (2023)	Lung	Mice: Ortho	40 d	CO gas 250 ppm Inhalation	No	30 min twice daily (d7 to d40)	Less graft injury with lower immune cell infiltration, fibrosis, airway obliteration, and total collagen	[64]
Sener (2013)	Kidney	Rat: Ortho	12 d	CORM-3, 100 μmol/L in UW	For 26 h after procurement	No	Less graft injury and improved graft survival via anti-apoptosis effect	[65]
Abe (2017)	Kidney	Rat: Ortho	100 d	High pressure chamber 2000 hPa	For 1 d after procurement	No	Less graft injury via less oxidative stress and pro-inflammatory cytokine mRNA expression, accompanied by activation of PI3K/Akt and p38 MAPK signaling pathways	[66]

Abbreviations: CORM: carbon monoxide-releasing molecules; CO: carbon monoxide; d: day/days; h: hour/hours; Hetero: heterotopic; hPa: hectopascal; IP: intraperitoneal; min: minutes; Ortho: orthotopic; PI3K/Akt: phosphatidylinositol 3 kinase/protein kinase B; p38 MAPK: p38 mitogen-activated protein kinase; Tx: transplantation; UW: University of Wisconsin.

**Table 3:** Application of CO in the transplantation field based on porcine IRI model.

Author (Year)	Target Organ	Ischemia Model	Observation Period	Delivery Method	CO Duration	Main Effects	Ref.
Lavitrano (2004)	Heart	2-h cardiac arrest	1 h after reperfusion	CO gas 250 ppm Inhalation	2 h before ischemia	Less interstitial edema and cardiomyocytes apoptosis Higher ATP and phosphocreatine Required fewer defibrillations to restart the heart after cardioplegia	[85]
Ahlström (2009)	Heart	40 min coronary artery occlusion	During ischemia	CO gas 5% COH Inhalation	2 h before ischemia	Lower lactate level Less decreased glucose level	[86]
Ahlström (2011)	Heart	45 min coronary artery occlusion	1 h after reperfusion	CO gas 5% COHb concentration Inhalation	2 h before ischemia	No difference in lactate, glucose, or pyruvate	[87]
Iqbal (2021)	Heart	60 min coronary artery occlusion	7 d after reperfusion	CORM-A1 4.27 mM at 1ml/min IV	1 h starting at 15 min after ischemia	Lower absolute infarct area Better recovery of left ventricular function Lower biochemical myocardial injury Less cell proliferation and inflammation	[88]
Sahara (2010)	Lung	90 min pulmonary vessels clamp	56 d after reperfusion	CO gas 250 ppm Inhalation	6 h until 2 h after reperfusion	Higher arterial oxygen concentration Lower inflammatory cell infiltration and cytokine level Fewer changes on chest x-ray and less pathological injury	[89]
Goebel (2011)	Lung	120 min cardiopulmonary bypass	5 h after reperfusion	CO gas 250 ppm Inhalation	1 h after cardiopulmonary bypass	Less alveolar edema, atelectasis, and inflammatory cell infiltration and cytokines Increased HSP70 and IL-10 levels	[90]
Bagul (2008)	Kidney	10 min warm and 18-h cold ischemia	3 h after reperfusion	CORM-3 50, 100, 200, or 400 µM in perfusion	1 h after reperfusion	50, 100 µM: Improved renal blood flow and function 200 and 400 µM: Poor renal hemodynamics and function	[91]
Hosgood (2008)	Kidney	10 min warm and 16 h cold ischemia plus 2 h NMP	3 h after reperfusion (Ex-vivo evaluation)	CORM-3 50 µM in perfusion	2 h during NMP	Improved renal blood flow and function	[92]
Bhattacharjee (2018)	Kidney	1 h warm and 4 h HMP	10 h after reperfusion (Ex vivo evaluation)	CORM-401, 200 µM in perfusate	20 min after HMP	Improved renal function and less urine protein excretion Less pathological injury Less vascular clotting	[93]
Murokawa (2020)	Liver	45 min portal vein and hepatic artery clamp	30 d	CO gas 250 ppm Inhalation	345 min until 2 h after reperfusion	Improved liver function Less pathological injury Lower inflammatory cytokines	[94]

Abbreviations: CO: carbon monoxide; COHb: carboxyhemoglobin; CORM: carbon monoxide-releasing molecules; d: day/days; h: hour/hours; HMP: hypothermic machine perfusion; hPa: hectopascal; IL-10: interleukin-10; IRI: ischemia–reperfusion injury; IV: intravenous; min: minutes; NMP: normothermic machine perfusion.

**Table 4:** Application of CO in the transplantation field based on porcine transplant model.

Author (Year)	Target Organ	Tx Model	Observation Period	Delivery Method	CO for Donor	CO for Recipient	Main Effects	Ref.
Sahara (2010)	Lung	Allo Tx	Until graft loss	CO gas 200–250 ppm Inhalation	For 3h during Tx	For 390 min during Tx	Improved graft survival Delayed development of anti-donor antibodies Lower inflammatory cytokines	[80]
Sahara (2018)	Lung	Xeno Tx (to cynomolgus monkey)	Until graft loss	CO gas 200–250 ppm Inhalation	For 3h during Tx	For 6 h during Tx	Did not prolong overall xenograft survival Less platelet depletion and lower inflammatory cytokines Less macrophage and neutrophil infiltration	[95]
Hanto (2010)	Kidney	Allo Tx	7 d	Inhalation 2–3 mg/kg	No	For 1 h from initiation of Tx	Improved renal function and pathological renal injury Less pro-inflammatory gene expression	[96]
Yoshida (2010)	Kidney	Autologous Tx	17 d	CO gas 5–10% In UW solution	For 2 d during preservation	No	Improved renal function, survival and pathological renal injury Lower inflammatory cytokines	[97]

Abbreviations: CO: carbon monoxide; d: days; h: hour/hours; Tx: transplantation; UW: University of Wisconsin; Xeno: xenotransplantation.

**Table 5:** Comparison among CO, NO, and H_2_S.

Formula	CO	NO	H_2_S
Color and odor	Colorless, odorless	Colorless, sweet odor	Colorless, rotten egg odor
Toxicity	High	High	High
Lipophilicity	Moderate	Low	High
Substrate	Heme proteins	L-arginine	L-cysteine
Biosynthetic enzymes	HO-1, HO-2	eNOS, nNOS	CBS, CSE, 3-MST
Delivery method	Inhalation, CO-releasing molecules	Inhalation, NO-releasing compounds	H_2_S donors
Measurement method	CO-oximeter	Chemiluminescence, fluorescence, MRI	MB spectrophotometric, S2- ion electrodes
Vasoregulatory activity	Vasodilation	Potent vasodilation	Vasodilation
Anti-inflammatory and anti-apoptotic effects	Yes	Yes	Yes
Therapeutic application	Alleviation of inflammatory injury in the circulatory system	Treatment of acute respiratory distress syndrome	Amelioration of renal fibrosis and dysfunction
	Protection of respiratory and digestive organs	Protection against excitotoxicity and neural modulation	Cardiovascular protection and prevention of CVDs
	Improvement in IRI and transplantation outcomes	Potential application in kidney transplantation	Neuroprotection and enhancement of cognitive function
			Modulation of cancer progression and anticancer effects

Abbreviations: CBS: cystathionine β-synthase; CSE: cystathionine γ-lyase; CO: carbon monoxide; CVDs: cardiovascular diseases; d: days; eNOS: endothelial nitric oxide synthase; h: hour/hours; H_2_S: hydrogen sulfide; HO-1: heme oxygenase-1; HO-2: heme oxygenase-2; IRI: ischemia–reperfusion injury; MPST: mercaptopyruvate sulfurtransferase; MRI: magnetic resonance imaging; NO: nitric oxide; nNOS: neuronal nitric oxide synthase; PPAR: peroxisome proliferator-activated receptor; PI3K/Akt: phosphatidylinositol 3-kinase/protein kinase B; p38 MAPK: p38 mitogen-activated protein kinase; Xeno: xenotransplantation.

## Data Availability

Dataset available on request from the authors.

## Abbreviations

The following abbreviations are used in this manuscript:

Adora1	Adenosine A1 receptor
A2A/A2B	Adenosine A2A/A2B receptors
Apaf-1	Apoptotic protease activating factor 1
ATP	Adenosine triphosphate
BAK	BCL2 antagonist/killer
BAX	BCL2-associated X protein
BCL2	B-cell CLL/lymphoma 2
CD39	Cluster of differentiation 39
cGMP	Cyclic guanosine monophosphate
CO	Carbon monoxide
COHb	Carboxyhemoglobin
COX	Cyclooxygenase
CORM	Carbon monoxide-releasing molecule
CytOx	Cytochrome c oxidase
DAMP	Damage-associated molecular pattern
eNOS	Endothelial nitric oxide synthase
ERK	Extracellular signal-regulated kinase
FasL	Fas ligand
Fe	Iron
GSK3β	Glycogen synthase kinase 3 beta
H_2_O_2_	Hydrogen peroxide
H_2_S	Hydrogen sulfide
HIF-1	Hypoxia-inducible factor 1
HMGB1	High-mobility group box 1
Hsp70	Heat shock protein 70
HO	Heme oxygenase
IL	Interleukin
IFN-γ	Interferon gamma
IRI	Ischemia–reperfusion injury
JAK	Janus kinase
JNK	c-Jun N-terminal kinase
KIM-1	Kidney injury molecule-1
LPS	Lipopolysaccharide
MAPK	Mitogen-activated protein kinase
MIP-1β	Macrophage inflammatory protein-1 beta
MMP	Matrix metalloproteinase
NADPH	Nicotinamide adenine dinucleotide phosphate
NO	Nitric oxide
NOX	NADPH oxidase
nNOS	Neuronal nitric oxide synthase
PPAR	Peroxisome proliferator-activated receptor
PI3K/Akt	Phosphoinositide 3-kinase/protein kinase B
PolyPHb	Polymerized human placenta hemoglobin
RBC	Red blood cell
ROS	Reactive oxygen species
SMA	Smooth muscle actin
STAT	Signal transducer and activator of transcription
SIRT1	Sirtuin 1
TGF-β1	Transforming growth factor beta 1
TNF-α	Tumor necrosis factor alpha
Tx	Transplantation
UW	University of Wisconsin
Xeno	Xenotransplantation
sGC	Soluble guanylate cyclase
